# New precipitation method for isolation of ^99m^Tc from irradiated ^100^Mo target

**DOI:** 10.1007/s10967-016-4967-2

**Published:** 2016-08-04

**Authors:** M. Gumiela, J. Dudek, A. Bilewicz

**Affiliations:** Institute of Nuclear Chemistry and Technology, Dorodna 16, 03-195 Warsaw, Poland

**Keywords:** *AnaLigTc*-*02*, Cyclotron, ^99m^Tc, Ammonium molybdenum phosphate

## Abstract

Technetium-99m is the most widely used radionuclide in nuclear medicine. This work describes the method to separate ^99m^Tc from irradiated ^100^Mo target. For this purpose we utilized formation of ammonium molybdenum phosphate (AMP) and have optimized the four parameters of the process. The proposed process is promising and allows fast separation of macroamounts of molybdenum without co-precipitation of ^99m^Tc. The concentration of molybdenum in solution after precipitation of AMP was lower than 300 µg ml^−1^. Additional purification using *AnaLigTc*-*02* is required to obtain solution with lower concentration of molybdenum.

## Introduction

Radionuclide ^99m^Tc is an ideal gamma emitter, because of its favorable half-life, photon energy, and versatile chemistry. It is annually used in about 30 million medical diagnostic procedures throughout the world [1]. Till now the ^99^Mo/^99m^Tc generator remains as the main source of ^99m^Tc production for nuclear medicine. The fragility of ^99^Mo supply for ^99^Mo/^99m^Tc generator production was highlighted by recent shutdown at two of the leading production sites for ^99^Mo. The Canadian Chalk River nuclear reactor, which supplies 35–40 % of the global consumption of ^99^Mo, will terminate its isotope production service in 2016 [2]. Other reactors supplying ^99^Mo are relatively old and are at the risk of prolonged or permanent shutdown within a few years, creating a risk of losing a long-term, stable supply of ^99^Mo for medical purposes. A variety of alternative options including both reactor and accelerator paths are evolving for sustainable production of ^99^Mo or ^99m^Tc directly for clinical use [3]. Reactor method of ^98^Mo thermal neutron irradiation produces low specific activity ^99^Mo through the ^98^Mo (n,γ)^99^Mo transformation [4]. Accelerator-based production of ^99^Mo, through the ^235^U (γ,f)^99^Mo and ^100^Mo (γ,n)^99^Mo reactions [5, 6] was recently elaborated and these methods make it possible to obtain high activity ^99^Mo. Currently, due to the large number of cyclotrons in the world, the most promising method is irradiation of enriched ^100^Mo by protons in the ^100^Mo (p,2n)^99m^Tc reaction. The method was identified almost 40 years ago [7] and its production parameters have since been investigated using a wide range of cyclotrons [8, 9]. It is possible to produce large quantities of ^99m^Tc using proton beam with energy of 16 MeV which is accessible in hundreds of medical cyclotrons over the world.

Extraction of technetium from irradiated molybdenum target can be carried out using either “wet” or “dry” chemical processes. Dry thermochromatographic system requires heating of the target under controlled atmosphere in a quartz tube [10, 11]. Due to the temperature gradient in the tube, and higher vapour pressure of the technetium species, separation is achieved when the two species are adsorbed at different locations on the quartz tube wall. Wet separation techniques require oxidative dissolution of the target. Separation of trace level of ^99m^Tc pertechnetate from macroamount of molybdate can be reached using one of many methods (e.g. liquid–liquid extraction [12, 13], ion-exchange chromatography [14], aqueous biphasic extraction chromatography, ABEC™ [15] and the electrochemical method [16]. Enriched molybdenum (>95 % ^100^Mo) currently costs between $0.85 and $3.00 per mg. Depending on the irradiation conditions up to a gram of material may be needed for each cyclotron target. Recycling of the molybdenum material is an important aspect in the economic viability of cyclotron production of ^99m^Tc. Therefore, the proposed method must be able to receive pure molybdenum with no impurities from other elements, that could be activated in the next irradiations.

In our work we decided to develop and use a different new simple and wet chemistry method of obtaining ^99m^Tc from the ^100^Mo target, containing in the first step isolation of molybdenum by precipitation of ammonium molybdenum phosphate. This method gives opportunity to obtain high yield of ^100^Mo recovery and perform the radiochemical separation as fast as possible.

## Experimental

### Reagents

All chemicals were analytically pure grade and used without further purification. The solutions were prepared in deionized water with electrical conductivity lower than 10 μS cm^−1^ at 25 °C (Millipore, Direct-Q3). Molybdenum powder *d* < 150 μm, 99.99 %, and triammonium phosphate trihydrate were purchased form Sigma Aldrich. Nitric acid 65 %, sodium hydroxide and ammonium nitrate was purchased from POCH Gliwice. 0.9 % sodium chloride solution was purchased from Sigma Aldrich. Polyethylene glycol 2000 (PEG-2000) was obtained from Merck and Cartridges Oasis HLB 6 cc was purchased from Waters. ^99m^Tc used in the optimization of the ion separation process was obtained from standard elution of an expired ^99^Mo/^99m^Tc generator supplied by Polatom. *AnaLigTc*-*02* (60–100 mesh) was purchsed from IBC Advanced Technologies. Dowex 50WX2 (100–200 mesh) was bought from Laboratorium Reagenzien, Heidelberg.

### Measurements

Radioactivity measurements were carried out using a calibrated intrinsic Ge detector (crystal active volume 100 cm^3^) and PC-based Multichannel Analyzer (MCA, Canberra). The detector had a resolution of 0.8 at 5.9, 1.0 at 123, and 1.9 at 1,332 keV. The 141 keV gamma-line was used. Molybdenum concentrations were determined by flame AAS (AAS Solaar M6 Thermo Electron England). The ICP-MS instrument, ELAN DRC II PerkinElmerTM, with a cross-flow nebulizer and with a Scott double-pass spray chamber and Ni cones was used in measurements.

### Chemical reprocessing

**Fig. 1:**
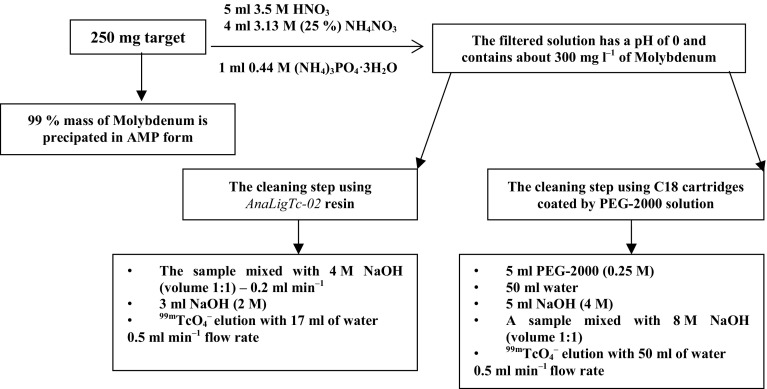
Separation scheme of the process

In order to find optimal separation conditions experiments were performed using surrogates. The metallic Mo powder was dissolved in a minimum volume of 3.5 M HNO_3_ and about 100 MBq of 99mTc from generator was added. Next, triammonium phosphate and ammonium nitrate were added to the solution and Mo was precipitated in the form of a yellow solid. After filtration of the solid the solution containing ^99m^Tc and trace impurities of Mo was alkalized by 8 M NaOH to get 4 M solution of NaOH. The alkalized solution was passed through C18 cartridges (Oasis HLB Plus 225 mg) coated by PEG-2000. After loading the column was washed with 5 ml of 4 M NaOH and ^99m^TcO_4_^–^ was eluted using 50 ml of water with flow rate 0.5 ml min^−1^. In the second method we used a column filled with *AnaLigTc*-*02*. In this approach 8.5 ml of solution was mixed after precipitation with the same volume of 4 M NaOH and with 200 μl of ^99m^TcO_4_^–^ which was eluted form generator. The alkalized solution was passed through the column filled with *AnaLigTc*-*02* resin. After loading the column was washed with 3 ml of 2 M NaOH and ^99m^TcO_4_^–^ was eluted using 17 ml of water with flow rate 0.5 ml min^−1^. Using Dowex-50WX2 ion exchange resin ions Na^+^ are removed and replaced by H^+^. For this purpose a glass column was filled with 2 g of Dowex-50WX2 resin which had been previously washed with water. The sorbent was conditioned with 15 ml of 2 M HCl. Next, eluent containing ^99m^Tc was passed through this column. After this, solution was directed to the top of Al_2_O_3_ column. A glass column was filled with 1 g of the alumina suspended in 0.01 M HNO_3_. The ^99m^TcO_4_^–^ was eluted with 0.9 % NaCl. Flow rate was maintained at 1 ml min^−1^. The schematic diagram of the process is presented in Fig. 1.

### Irradiation of Mo target by protons

After establishing optimal conditions for separation of ^99m^Tc from macroamounts of molybdenum we tested our method on the irradiated metallic molybdenum target. In order to prepare the molybdenum target material for its irradiation with protons, molybdenum powder was pressed into pellets followed by its sintering. The powdered molybdenum was pressed for 60–90 min by the use of hydraulic press PLH-25, enabling to obtain pressure inside the matrix from 800 to 1000 MPa. The diameter of the pellet was 14 mm, thickness 0.720 mm and density 9.6 g cm^−3^. The pellet was loaded into aluminum holder which was mounted in GE PETtrace 840 cyclotron (at Heavy Ion Laboratory, University of Warsaw). The target was irradiated for 15 min in the external, well cooled target holder with proton beam at 2 µA current up to total activity of 110 MBq at the end of bombarding. The target was automatically disassembled into transportation container and send for reprocessing using procedure elaborated for simulated solution.

## Results

The idea of our studies is the preliminary separation of bulk molybdenum in the process of precipitation ammonium molybdenum phosphate (AMP). AMP is the inorganic salt of phosphomolybdic acid with the chemical formula (NH_4_)_3_PMo_12_O_40_. It is formed in solution according to the reaction (1):

1 $$\left( {{\text{NH}}_{4} } \right)_{3} {\text{PO}}_{4} \cdot 3 {\text{H}}_{2} {\text{O}} + 1 2 {\text{MoO}}_{4}^{2 - } + 2 4 {\text{H}}^{ + } \to \left( {{\text{NH}}_{4} } \right)_{3} {\text{PMo}}_{12} {\text{O}}_{40} + 1 5 {\text{H}}_{2} {\text{O}}.$$

Literature data indicate that solubility of AMP in acidic solution is very low—137 mg in 100 ml 5 % of NH_4_NO_3_ solution [17]. Such low solubility of AMP should allow preliminary separation of molybdenum from the solution.

### Dissolution of Mo metallic target

To dissolve metallic molybdenum 3.5 M HNO_3_ was chosen. After few minutes metallic Mo dissolved completely. The HNO_3_ concentration of 3.5 M is optimum because in higher concentration of HNO_3_ hydrolysis of AMP is occurred and the concentration of Mo in solution increases.

### Separation of molybdenum by precipitation of AMP

In order to find optimum conditions for separation of molybdenum by precipitation of AMP the influence of triammonium phosphate and ammonium nitrate concentration was studied. The results are presented in Tables 1 and 2. This part of experiment was carried out at 60 °C.

**Table 1:** Influence of triammonium phosphate (NH_4_)_3_PO_4_·3H_2_O concentration on efficiency of Mo precipitation

Concentration of triammonium phosphate trihydrate (M)	Excess of stoichiometric amount of (NH_4_)_3_PO_4_·3H_2_O to MoO_4_^2−^ (%)	Concentration of Mo in solution (mg ml^−1^)	% Mo in precipitate
0.22	0	2.26 ± 0.11	90.96 ± 0.45
0.44	100	1.25 ± 0.00	95.00 ± 0.00
0.77	250	1.14 ± 0.15	95.46 ± 0.59

**Table 2:** Influence of NH_4_NO_3_ addition on Mo separation from solution

Concentration of ammonium nitrate (M)	Concentration of Mo in solution (mg ml^−1^)	% Mo in precipitate
0	1.25 ± 0	95.0 ± 0.0
0.13	1.05 ± 0.04	95.8 ± 0.2
0.31	0.94 ± 0.02	96.2 ± 0.1
1.25	0.69 ± 0.05	97.3 ± 0.2
3.13	0.57 ± 0,01	97.7 ± 0.1

As shown in Table 1 in order to effectively precipitate molybdenum twofold excess of triammonium phosphate is sufficient. We have also found that further addition of (NH_4_)_3_PO_4_·3H_2_O does not influence the AMP solubility. To improve the efficiency of the process we have examined the effect of ammonium nitrate addition on the yield of AMP precipitation, see Table 2.

**Fig. 2:**
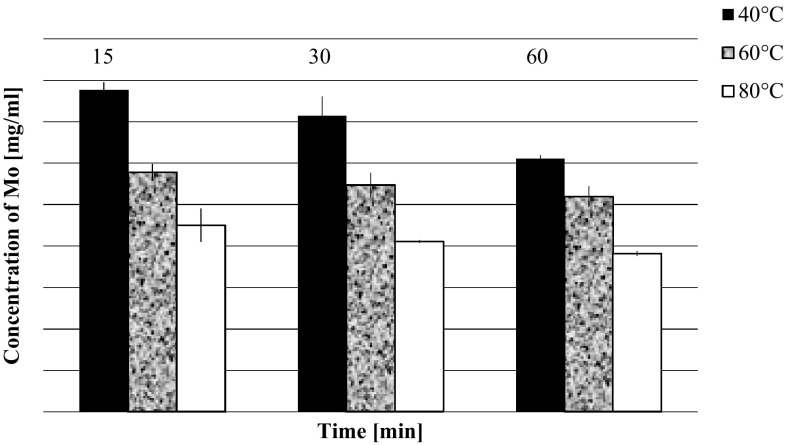
Concentration of Mo in solution as a function of time and temperature of the precipitation process

As can be seen in Table 2 addition of ammonium nitrate (3.13 M) increased precipitation of AMP from 95.0 to 97.7 % In 3.13 M ammonium nitrate solution the solubility of AMP is only half of that when ammonium nitrate is absent. Influence of temperature and time on precipitation have been also investigated. As one can see in Fig. 2 the concentration of Mo in the reaction mixture only slightly depends on the time of sample heating. The lowest concentration of Mo in the solution, equal to about 370 µg ml^−1^, was reached when the reaction mixture was heated to 80 °C.

Based on the performed experiments the following parameters of AMP precipitation were selected. To 250 mg of metallic Mo target dissolved in 5 ml 3.5 M HNO_3_ 0.77 mmol of (NH_4_)_3_PO_4_ and 12 mmol of NH_4_NO_3_ were added. The precipitate was separated after 15 min by filtration or centrifugation. Under these conditions the concentration of Mo in the aqueous solution decreased from 25 to 0.3 mg ml^−1^.

An important parameter in the separation process involving precipitation method is the ability of the separated substances to co-precipitate. Because the concentration of ^99m^TcO_4_^–^ in solution is 10^7^ times smaller than that of Mo the possibility of coprecipitation of ^99m^Tc with AMP can be significant. However, we found that coprecipitation was negligible and after separation 99 % of total ^99m^Tc activity remained in the solution.

### Purification using PEG-2000 or *AnaLigTc*-*02* resin

**Fig. 3:**
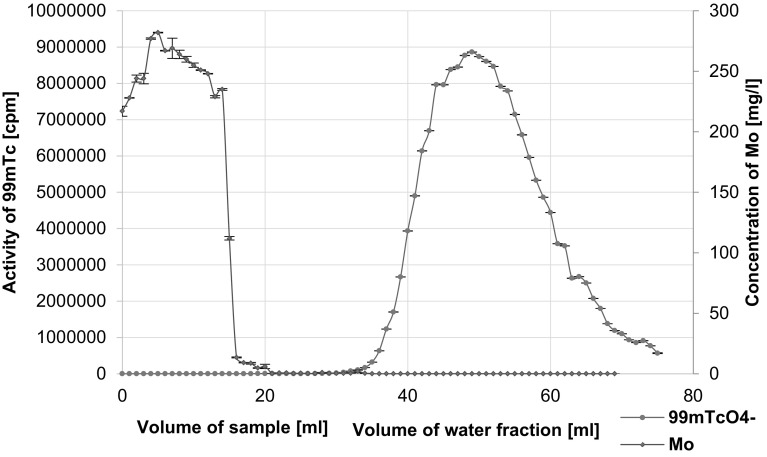
Elution profile of Mo and ^99m^Tc from C-18 column coated with PEG 2000

After filtration of AMP the solution contains about 0.3 mg ml^−1^ of molybdenum. Because the accepted value for Mo in ^99m^Tc radiopharmaceuticals cannot exceed 10 ppm, additional process of purification is needed. In order to remove the residue of Mo we have used PEG-2000 modified sorbents, in which only pertechnetate anions are adsorbed from high concentration ionic solutions (4 M NaOH) and can be eluted using pure water. This procedure was previously described [18] except that now KOH was replaced by NaOH. As shown in Fig. 3 application of C-18 cartridge modified with polyethylene glycol allowed for complete separation of ^99m^Tc from Mo with recovery of ^99m^Tc greater than 70 %. We did not find any trace of Mo in ^99m^Tc fractions. Unfortunately ^99m^TcO_4_^–^ was eluted by relatively large (about 50 ml) volume of water so that a preconcentration process was needed.

**Fig. 4:**
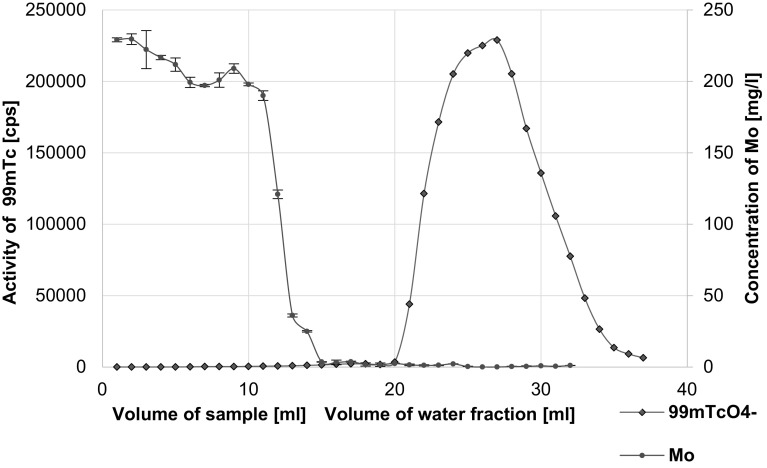
Elution profile of Mo and ^99m^Tc in the case of *AnaLigTc*-*02* resin

In order to remove the residue of Mo we used also the *AnaLigTc*-*02* resin, on which the pertechnetate anions are adsorbed from high concentration ionic solutions and ^99m^TcO_4_^–^ anions are eluted using distilled water. The elution profile is shown in Fig. 4.

Comparison of the two profiles and taking into account the duration of the processes and recovery of pertechnetate, when we applied the specific *AnaLigTc*-*02* resin, we got better results, see Table 3.

**Table 3:** Comparison C-18 column coated with PEG 2000 with *AnaLigTc*-*02* resin

	Cartridges oasis HLB 6 cc coated with PEG 2000	*AnaLigTc*-*02* resin
Volume of water fraction with ^99m^TcO_4_^−^ (ml)	50	17
The recovery of ^99m^TcO_4_^−^ (%)	71.6	95.8
Duration (min)	170	130

### Preconcentration ^99m^Tc fraction

**Fig. 5:**
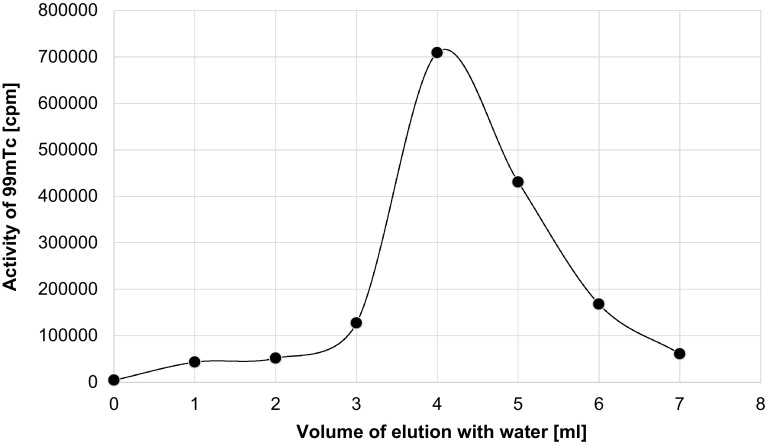
Elution profile of ^99m^Tc from alumina column

The eluate of ^99m^Tc recovered from the C-18 PEG 2000 column was slightly alkaline. Therefore before passing through alumina column 17 ml of the ^99m^Tc solution was neutralized in the cation exchange resin (Dowex-50WX2) and next trapped on a small alumina column. The ^99m^TcO_4_^–^ was eluted with 0.9 % NaCl (Fig. 5). This preconcentration process is very efficient because ^99m^TcO_4_^–^ is eluted with 7 ml of 0.9 % NaCl with nearly 100 % of recovery. Concentration of Mo in this fraction is 0.40 ppm, which indicates that the product obtained is chemically pure.

### Reprocessing of cyclotron irradiated Mo target

To verify the proposed procedure the separation process of ^99m^Tc described above was tested on the protons irradiated molybdenum target. The target after releasing from the holder was dissolved in 3.5 M HNO_3_ and the separation procedure elaborated for simulated solution was applied. Due to shorter time of separation we used column filled with *AnaLigTc*-*02* resin. The percent recovery of ^99m^Tc in each step of separation process is presented in Table 4.

**Table 4:** The balance of ^99m^Tc radioactivity in the separation process

Separation step	% recovery of ^99m^Tc
Precipitation of AMP	93.1
Purification on *AnaLigTc*-*02* resin	59.2
Preconcentration on Al_2_O_3_ bed	99.0

It can be seen in Table 4. Taking into account ^99m^Tc decay during the separation process total ^99m^Tc recovery yield was equal to 33.6 %. The largest loss of ^99m^Tc was observed during the purification process on Analig bed. In model experiments, when generator produced ^99m^Tc was used, purification on Analig bed exceeded 90 %. Lower recovery of ^99m^Tc in the case of cyclotron irradiated target is probably associated with incomplete transition of technetium to pertechnetate and formation of TcO_2_ colloid. The obtained ^99m^Tc solution was free of molybdenum and was ready for labelling.

## Conclusions

We have presented in this paper for the first time an alternative precipitation method for separation of ^99m^Tc from protons irradiated Mo target. The proposed method is fast and simple and gives in model experiments the recovery of ^99m^Tc greater than 96 %. Unfortunately, in the process where irradiated target was used the recovery of ^99m^Tc from the irradiated ^100^Mo recovery decrease to 50 %. Therefore, further improvement of the purification process is necessary. As tested on HYNIC-substance–P labelling the resulting saline-pertechnetate solution closely matches the product obtained from the ^99^Mo/^99m^Tc generator.

Molybdenum target material can be easily recovered from the waste stream as AMP. More than 90 % of Mo goes to the AMP. This is an important aspect of using enriched molybdenum as the target material, because recycling of the expensive stable isotope will be cost-effective. As shown previously [19] AMP can be thermally decomposed in the range of 680–753 K according to the reaction Eq (2):

2 $$8 \left[ {\left( {{\text{NH}}_{4} } \right)_{3} {\text{PO}}_{4} \cdot 1 2 {\text{MoO}}_{3} } \right]\mathop{\longrightarrow}\limits^{temp.} 8 \left[ {{\text{H}}_{3} {\text{PO}}_{4} \cdot 1 2 {\text{MoO}}_{3} } \right] + 2 4 {\text{NH}}_{3} .$$

The formed phosphoric acid which can be easy separated by washing with water and formed MoO_3_ will be next reduced to Mo metal by hydrogen. The advantage of this methods is isolation of Mo target material in the first step of the process, because the next stages of separation use metals cations like Na^+^ which are easily activated in irradiation process. The proposed methodology may be also applied to the cyclotron production of other medically relevant technetium isotopes (e.g. ^94m^Tc). The recovery of Molybdenum from AMP is the subject of further research.
